# Combined analysis of lipidomics and transcriptomics revealed the key pathways and genes of lipids in light-sensitive albino tea plant (*Camellia sinensis* cv. *Baijiguan*)

**DOI:** 10.3389/fpls.2022.1035119

**Published:** 2022-10-18

**Authors:** Zhe Zhou, Mingjie Chen, Quanjin Wu, Wen Zeng, Zhidan Chen, Weijiang Sun

**Affiliations:** ^1^ College of Horticulture, Fujian Agriculture and Forestry University, Fuzhou, China; ^2^ Ministerial and Provincial Joint Innovation Centre for Safety Production of Cross-Strait Crops, Fujian Agriculture and Forestry University, Fuzhou, China; ^3^ College of Life Science, Xinyang Normal University, Xinyang, China; ^4^ Department of Finance and Management, The Open University of Fujian, Fuzhou, China; ^5^ Anxi College of Tea Science, Fujian Agriculture and Forestry University, Quanzhou, China

**Keywords:** *Camellia sinensis*, albino tea plant, light, lipidomics, transcriptomics, WGCNA

## Abstract

Currently, the mechanism by which light-sensitive albino tea plants respond to light to regulate pigment synthesis has been only partially elucidated. However, few studies have focused on the role of lipid metabolism in the whitening of tea leaves. Therefore, in our study, the leaves of the *Baijiguan* (BJG) tea tree under shade and light restoration conditions were analyzed by a combination of lipidomics and transcriptomics. The leaf color of BJG was regulated by light intensity and responded to light changes in light by altering the contents and proportions of lipids. According to the correlation analysis, we found three key lipid components that were significantly associated with the chlorophyll SPAD value, namely, MGDG (36:6), DGDG (36:6) and DGDG (34:3). Further weighted gene coexpression network analysis (WGCNA) showed that *HY5* TF and *GLIP* genes may be hub genes involved lipid regulation in albino tea leaves. Our results lay a foundation for further exploration of the color changes in albino tea leaves.

## Introduction

The tea plant (*Camellia sinensis*) is a perennial evergreen woody plant, of which its shoots are often processed into various tea products, such as green tea, black tea, and oolong tea (Li et al., 2019). To date, tea cultivars and their products in China show a ‘colorful’ trend (Wang et al., 2015). Albino tea cultivars are special mutants of the tea plants with white or yellow leaves under certain environmental conditions, such as low temperature or high light intensity (Du et al., 2006; Peng et al., 2012; Feng et al., 2014). Compared to normal green cultivars, albino cultivars are deficient in chlorophyll. The albino tea germplasm is valuable because of its special flavor, distinct leaf color and scarcity (Du et al., 2006). Sunlight is one of the necessary conditions for photosynthesis and plays an important role in the chlorophyll synthesis (Fan et al., 2019). BJG, a light-sensitive albino tea cultivar, the new shoots display a white color under high light intensity, and turn green under low light intensity (Wu et al., 2016). Currently, the research on the formation of albino leaves is mainly focused on the metabolism of chlorophyll and carotenoids (Wang et al., 2014; Wang et al., 2016; Liu et al., 2017a; Lu et al., 2019). Under sunlight, the chlorophyll synthesis in *Huangjinya* is blocked and its degradation is accelerated. The contents of the antenna protein and-PSII-and PSI-related proteins were significantly reduced, resulting in arrest of photosynthetic electron transport and a reduction in photosynthetic efficiency (Fan et al., 2019). Transcriptomic analysis found that the affected genes were enriched in the fatty acid metabolic pathway and unsaturated fatty acid metabolic pathway (Wu, 2015).

Lipids are the structural materials of cell membranes that play a number of key roles in plant growth, development, and responses to environmental factors (Welti and Wang, 2004; Liu et al., 2017b). The comprehensive classification system organizes lipids into eight well-defined categories: Fatty acyls, Glycerolipids, Glycerophospholipids, Sphingolipids, Sterol Lipids, Prenol Lipids, Saccharolipids and Polyketides (Fahy et al., 2009). The membranes of plant cells containing 5% to 10% lipids (dry weight) are able to distinguish the cells and compartments where many key processes occur, including the light harvesting and electron transport reactions of photosynthesis (Ohlrogge and Browse, 1995). The chloroplast thylakoid membrane is the place where plants perform photosynthesis. The lipid bilayer of the thylakoid membrane is mainly composed of monogalactosyldiacylglycerol (MGDG) and digalactosyldiacylglycerol (DGDG), which account for a large proportion, as well as sulfoquinovosyldiacylglycerol (SQDG) and phosphatidylglycerol (PG). The synthesis of fatty acids in chloroplast intermediates is the first step in the production of chloroplast lipids and is catalyzed by chloroplast FA synthase (FAS) and acetyl CoA carboxylase (ACC), while phosphatidylic acid (PA) can be produced in the chloroplast and endoplasmic reticulum (ER), depending on the plant species (Benning, 2009; Troncoso-Ponce et al., 2016; Li et al., 2020). Fatty acids must be transported from the plastids to the endoplasmic reticulum, where most of the *de novo* synthesized fatty acids assemble into phospholipids and neutral lipids in the endoplasmic reticulum (Li-Beisson et al., 2013). Fatty acids are synthesized by the condensation, dehydration and reduction of acyl carrier proteins, mainly through prokaryotic and eukaryotic pathways (Boudière et al., 2014; Li et al., 2016; Troncoso-Ponce et al., 2016; Hölzl and Dörmann, 2019). Fatty acids enter the ER and bind through two pathways, one of which is the Kennedy pathway. Fatty acids in the form of fatty acyl-CoA are catalyzed by glycerol-3-phosphate acyltransferase (GPAT) to produce lysophosphatidic acid (LPA). LPA is catalyzed by 1-acyl-sn-glycerol-3-phosphate acyltransferase (plsC) to generate phosphatidic acid (PA). Then, PA is phosphorylated by phosphatidylic acid phosphatase (PLPP) to generate diacylglycerols (DAG). DAG binds to various lipids to generate other lipids such as phosphatidylcholine (PC) (Chapman and Ohlrogge, 2012; LaBrant et al., 2018). The second pathway is “acyl editing”. In this pathway, fatty acids are directly added to lysophosphatidylcholine (LPC) to regenerate PC, which is recycled back into LPC. MGDG transfers galactose from uridine diphosphate galactose (UDP-Gal) to DAG backbone under the catalysis of MGDG synthetase (MGD). The second galactose is then transferred from UDP-Gal to MGDG by DGDG synthase (DGD) to form DGDG (Gigon et al., 2004; Lin et al., 2016). The photosynthetic protein complex in chloroplasts is embedded in polar lipids, which are regularly arranged and play a critical role in photosynthesis. The plasma membrane is considered as the main barrier between the organism and the external environment, and it is a material that can overcome pressure damage (Janik et al., 2013).

Changes in lipid content and proportion often alter the thylakoid membrane structure, which has been studied in other plants. Under low temperature, the thylakoid membrane structure becomes unstable, its development is not perfect or it even disintegrates, the stacking of basal grains is reduced, the lamellar structure is unclear, and the contents of starch grains increases. The contents of MGDG, DGDG, PG and other species of lipids, the MGDG to DGDG ratio, the contents of unsaturated fatty acids, and the membrane fluidity all are decreased, which ultimately affects the photosynthetic efficiency (Ling, 2001). Therefore, the changes in lipid composition and structure in the plasma membrane under environmental pressure play an important role in maintaining membrane stability and function. Tea plants can store triacylglycerol (TAG) in low nitrogen environments to maintain the C/N balance, which is helpful to improve the quality of tea. Under normal nitrogen treatment, TAG can ensure the transformation of the unique aroma of tea plants. However, after high nitrogen treatment, the increases of MGDG (36:6) and DGDG (36:6) will lead to an increase in aroma synthesis precursors and produce a large amount of grass gas, which is not conducive to improving of tea quality (Liu et al., 2017b). Under drought stress, the MGDG/DGDG ratio in tobacco was found to be increased, but the relative contents of C18:3 fatty acids in MGDG and DGDG decreased, which was not conducive to the stability of the thylakoid membrane (Wang et al., 2022). The lower photosynthetic rate of wheat under high temperature stress is the result of the interaction between thylakoid membrane damage, membrane lipid composition and organelle oxidative damage. High temperature stress during flowering significantly decrease the total amounts of MGDG, PG, PC and PA (Narayanan et al., 2016; Djanaguiraman et al., 2018; Djanaguiraman et al., 2020). Such an increase in unsaturated fatty acids in PG reduces the formation of ROS and the damage to photosynthetic complexes, thus improving the low temperature tolerance of tomato plants (Sun et al., 2011). In conclusion, abiotic stress can cause changes in the contents and proportions of chloroplast membrane lipids.

The issue of tea whitening has been widely studied. Among these studies, the gene expression profile, genetic structure, some specific base mutation sites and differential accumulation of secondary metabolites of photosensitive albino teas have been gradually clarified. Currently, we know that albino tea plants are mainly affected by the changes in photosensitive pigments in response to light signals, which induce the differential accumulation of tea pigment compounds. Especially in different light environments, the synthesis and regulation of these pigment compounds have been the focus of attention. Based on the clear response of tea plants to light stress, we understand that light management is a key regulator of albino tea plant leaf color phenotype. However, at the level of lipid metabolism, we know little about the photosensitive albino tea plants, and the correlation between its leaf color and lipid changes needs to be explored (Yue et al., 2021).

In this study, we analyzed the composition of lipids in the albino leaves of BJG and their changes in response to light through lipidomics in combination with transcriptomics to determine the key pathways and genes of that are involved. This work will help us to understand the discoloration mechanism of light-sensitive albino tea plants and allow us to make full use of the characteristics of albino tea plants to select excellent tea varieties.

## Materials and methods

### Plant materials and experiment

BJG was planted in the Wuyi Star Tea Germplasm Resources Garden at Wuyi Star Tea Co. LTD., Wuyi Mountain, China (27° 55 ′ 15 ″ N, 118° 02 ′ 50 ″ E). In the middle of September 2018, the second leaves of BJG with a similar growth and development status were taken for treatment. There were three treatments in the experiment: normal light (BS0), shading (BS) and shading for 3 days followed by the resuming of light treatment (BRL). The second leaf was covered with aluminum foil for shading treatment. After 3 days of shading, part of the aluminum foil was removed to restore the light to the blade. The total processing time was 6 days. The samples were named BS0, BS1, BS2, BS4, BS6, BRL1, and BRL3. Before plucking, the chlorophyll SPAD value was measured in the leaf. All samples were frozen with liquid nitrogen and stored at −80°C for lipidomics analysis. Among them, samples BS0, BS1 and BRL3 were also used for transcriptome sequencing.

### Determination of chlorophyll content

A SPAD-502PLUS Chl meter (Spectrum Technologies, Konica Minolta, Japan) was used to determine the chlorophyll SPAD value from the leaves (Uddling et al., 2007). We tested each leaf, avoiding the main vein, and repeated the test five times.

### Lipid isolation from tea leaves

Glass tubes or vials with Teflon-lined caps were used for all experiments. All solvents, including water, were HPLC-grade. Lipids were extracted from samples of BS0, BS1, BS4, BS6 and BRL3 according to the method of Shiva (Shiva et al., 2018). A total of 2.0 mL isopropanol/0.01% butylated hydroxytoluene was added into 8 mL glass tube with a screw cap (the exact weight of each empty tube exact weight was recorded), and the lid was closed snugly. Then, the mixture was heated to 75 °C. Six to eight tea leaves were rapidly harvested and put into hot isopropanol very quickly into hot isopropanol to avoid lipolytic activity. The lid was placed on snugly to prevent evaporation. The samples were heated for more than 15 minutes, and removed from the heating block and allowed to cool to room temperature. Next, 6.0 mL of chloroform/methanol/water (30/41.5/3.5, v/v/v) was added and the lid was closed snugly. The samples were shaken at room temperature for 24 h (50-100 rpm on shaker) until the green leaves became pale white, which indicated that the lipids had been completely extracted. The extracted tea leaves were transferred to a new vial using forceps, dried overnight at 105 °C, equilibrated to room temperature, and weighed in grams to 6 decimal places. Additionally, using a glass, gass-tight syringe (or a glass pipette), the solvent and lipids were transferred to a 2.0 ml GC vial (clear glass, Teflon lined screw cap lid) and dried down under a nitrogen stream before being taken to the Kansas Lipidomics Research Center (KLRC, USA) for lipid testing.

### Analysis of lipids by ESI-triple quadrupole MS in multiple reaction monitoring mode for the analysis of tea plants

These experiments were carried out referencing the methods of Shiva (Shiva et al., 2018) and Vu (Vu et al., 2014). An ESI-triple quadrupole mass spectrometry multiple reactions monitoring analysis method was used to detect lipids in tea plants. The data were processed using Lipidome DB Data Calculation Environment (http://129.237.137.125:8080/Lipidomics).

### Construction of a gene coexpression network module and determination of the enriched functions and pathways

Based on the transcriptome data of three samples, we screened genes with an average FPKM value greater than or equal to 1, and used the WGCNA R program to analyze the WGCNA (Langfelder and Horvath, 2008). First, it was assumed that the gene network obeys the scale-free distribution, and the correlation matrix of gene coexpression and the adjacency function formed by the gene network were defined. The optimal soft threshold in this investigation was power β = 20, and the resulting adjacency matrix was utilized to determine the topological overlap (TO). Then, the hierarchical clustering tree was constructed by using the dynamic hybrid tree cutting technology. We set the minimum module size to 30 and the minimum height of the combined module to 0.25. Naming genomes with different colors was found to be convenient for distinguishing different gene modules in the subsequent gene function recognition and visual analysis. According to the p value, GeneRatio and the number of differentially expressed genes annotated in the pathway, the KEGG pathway enrichment analysis of the key modules was carried out. Finally, the topological overlap measure from the WGCNA was displayed using Cytoscape 3.9.1 to illustrate the network.

### Statistical analysis

The mean and standard deviation of the chlorophyll SPAD value and lipid data were calculated using Microsoft Excel 2019. One-way analysis of variance (ANOVA) with Duncan’s test was performed using SPSS 22.0. Differential lipids were analyzed by MetaboAnalyst 5.0 (https://www.metaboanalyst.ca/). Principal component analysis (PCA), partial least-squares discriminant analysis (PLS-DA) were performed using SIMCA14.0. Heatmaps were generated using TBtools software. Correlation analysis was performed using Hiplot software (https://hiplot.com.cn). The correlation data network diagrams and coexpression network diagrams were generated using Cytoscape 3.8.2 software.

## Results

### Phenotypic characterization of BJG

**Figure 1:**
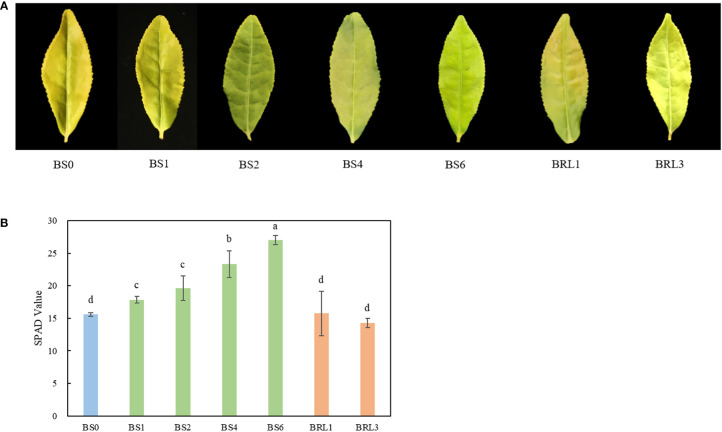
Leaf color phenotype and the SPAD value of BJG after shading and recovery light treatments. **(A)** Phenotypic expression and **(B)** the SPAD value of BJG under natural light (BS0), shading for 1 day (BS1), shading for 2 days (BS2), shading for 4 days (BS4), shading for 3 days then light recovery for 1day (BRL1), and shading for 3 days then light recovery for 3 days (BRL3). Data and error bars are the mean ± SD (n = 5). Different lowercase letters indicate a significant difference between the means at *P*<0.05.

We observed the second leaf of the BJG after different shading durations. Under normal conditions, the second leaf of BJG remained yellow. After shading treatment, the BJG leaf turned into green within one day, and this process could be reverted by again subjecting the leaves again to strong light (**Figure 1A**). Moreover, the chlorophyll SPAD value of the leaves under shading increased gradually and was significantly higher than that under light (*P*<0.05). However, the SPAD value after light restoration showed little change compared with that before shading (**Figure 1B**). The results showed that the shading treatment turned the leaves of BJG into green and increased the chlorophyll content.

### Overall lipidomics analysis of BJG after different shading duration

**Figure 2:**
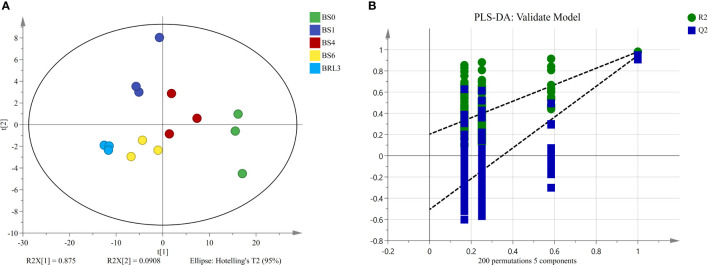
Unsupervised and supervised multivariate analysis of the detected lipids in BJG. **(A)** PCA score plot. **(B)** PLS-DA cross-validation plot.

According to the significant difference in chlorophyll SPAD values, we examined 5 samples for lipidomics detection and analysis. First, we conducted multivariate statistical analysis, which can provide preliminary insights into the overall differences between samples as well as variation between samples within groups. Clear differentiation in the samples was observed in the PCA score plot (**Figure 2A**). The cumulative contribution rates of the first (PC1) and second principal components (PC2) reached 96.58%, which indicated that the fit of the model was high and that the multidimensional statistical analysis results were reliable. Moreover, the cross-validation with 200 permutation tests indicated that the PLS-DA model was reliable and was not overfit (**Figure 2B**).

**Figure 3:**
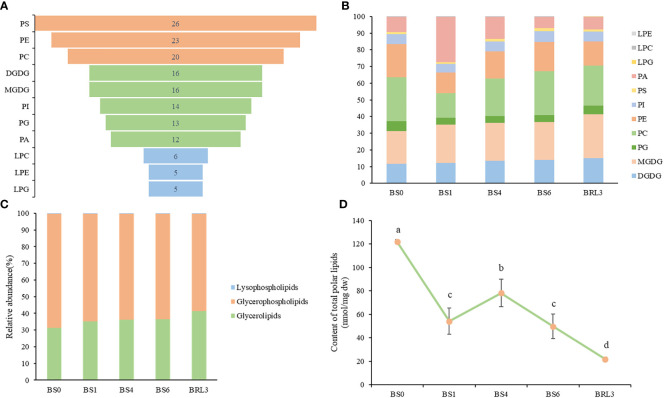
Overall lipidomics analysis of in BJG. **(A)** Distribution of each lipid subclass identified. **(B)** Changes of lipid subclasses in BJG after shading and light recovery treatments. **(C)** Lipid categories identified in BJG after shading and light recovery treatments. **(D)** Changes in the content of total polar lipids after shading and light recovery treatments. Data and error bars are the mean ± SD (n = 3). Different lowercase letters indicate a significant difference between the means at *P*<0.05.

Glycerolipids, including phospholipids, glycolipids and neutral lipids, are the most abundant lipids in plants. In this study, 156 lipid molecules were detected, including 16 DGDG, 16 MGDG, 13 PG, 5 lyso phosphatidylglycerols (LPG), 6 LPC, 5 lysophosphatidylethanolamine (LPE),20 PC, 23 phosphatidylethanolaines (PE), 14 phosphatidylinositiols (PI), 26 phosphatidylserines (PS) and 12 PA (**Figure 3A**). As shown in **Figure 3B**, lipids were classified into 3 lipid categories. The proportions of the different molecules and lipid classes in BJG were analyzed according to their relative contents. Among the lipid subclasses, PC and MGDG accounted for the largest proportion, and the lipids that showed large changes included PA, PE and PC. In BS1, the proportion of PC and PE decreased, while the proportions of PA increased (**Figure 3B**). Overall, glycerophospholipids accounted for the largest proportion, ranging from 58% to 68.3%, and the proportion after shading and light restoration was lower than that before shading (**Figure 3C**). Additionally, we calculated the content of total polar lipid content and found that it decreased after shading. Moreover, the content was the highest in BS0 and the lowest in BRL3 (**Figure 3D**).

### Glycolipid changes in BJG after shading and recovery light treatments

**Figure 4:**
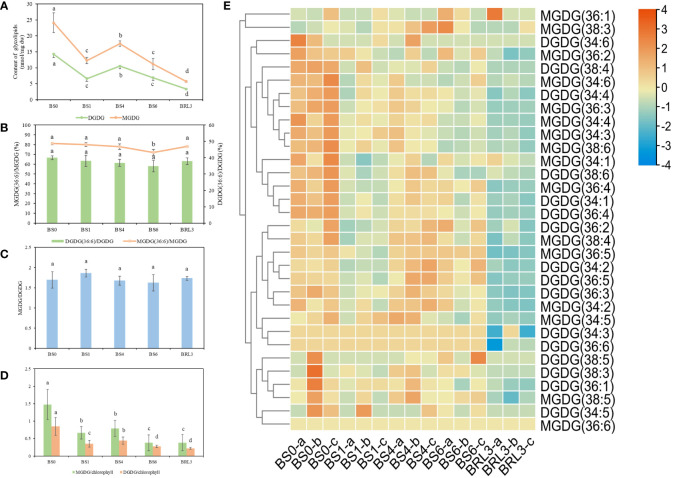
Changes in glycolipids in BJG after shading and light recovery treatments. **(A)** Changes in MGDG and DGDG contents in BJG after shading and light recovery treatments. **(B)** The ratio changes in the MGDG (36:6)/MGDG and DGDG (36:6)/DGDG ratios after shading and light recovery treatments. **(C)** The changes in the MGDG/DGDG ratio after shading and light recovery treatments. **(D)** The changes in the MGDG/chlorophyll and DGDG/chlorophyll ratios after shading and light recovery treatments. **(E)** Heatmap of glycolipid changes. Data and error bars are the mean ± SD (n = 3). Different lowercase letters indicate a significant difference between the means at *P*<0.05. Each colored cell on the map corresponds to the content of different lipid species. Orange color indicates a high content, and blue color indicates a low content.

MGDG and DGDG are the main components of the thylakoid membrane in plants. The changes in the contents of MGDG and DGDG in BJG showed the same trends as those of total lipids. Both lipids decreased sharply in BS1, then increased significantly in BS4, and decreased again in BS6. These lipids also showed a downward trend after returning to light exposure for 3 days (**Figures 4A, E**). MGDG and DGDG accounted for approximately 1/5 and 1/8 of the total lipids in BJG, respectively. As an important lipid molecule in tea, MGDG (36:6) accounted for the largest proportion in leaves. In BS0, its content was the highest, which reached 19.56 nmol/mg dw, and in BRL3, its content was the lowest at 4.47 nmol/mg dw. Similarly, among the DGDG species, DGDG (34:3) and DGDG (36:6), which are the main DGDG lipid molecules, have displayed their highest contents in BS0, which were 5.23 nmol/mg dw and 5.71 nmol/mg dw respectively. In BRL3, the lowest values were 1.27 nmol/mg dw and 1.25 nmol/mg dw, respectively (**Table S1**). By analyzing the ratios of MGDG (36:6)/MGDG and DGDG (36:6)/DGDG, we found that these ratios gradually decreased after shading and reached their lowest in BS6; These ratios then increased under light, and were highest in BS0 (**Figure 4B**).

The MGDG/DGDG ratio plays an important role in maintaining the structure and function of photosynthetic organs, which affects the permeability of the chloroplast membrane and the stability of the membrane bilayer (Block et al., 1983). The ratios of MGDG/DGDG in BS1 and BRL3 were higher than those in the other samples (**Figure 4C**). The membrane lipid/chlorophyll ratio is an indicator of the density of thylakoid assembly proteins. The higher the ratio is, the lower the density of the assembly protein is, which reflects the destruction of the fluidity, structure and function of the membrane (Haferkamp and Kirchhoff, 2008; Kirchhoff et al., 2013). The ratios of MGDG/chlorophyll and DGDG/chlorophyll decreased significantly as the number of treatment days increased (**Figure 4D**).

### Glycerophospholipid and lysophospholipid changes in BJG after shading and recovery light treatments

**Figure 5:**
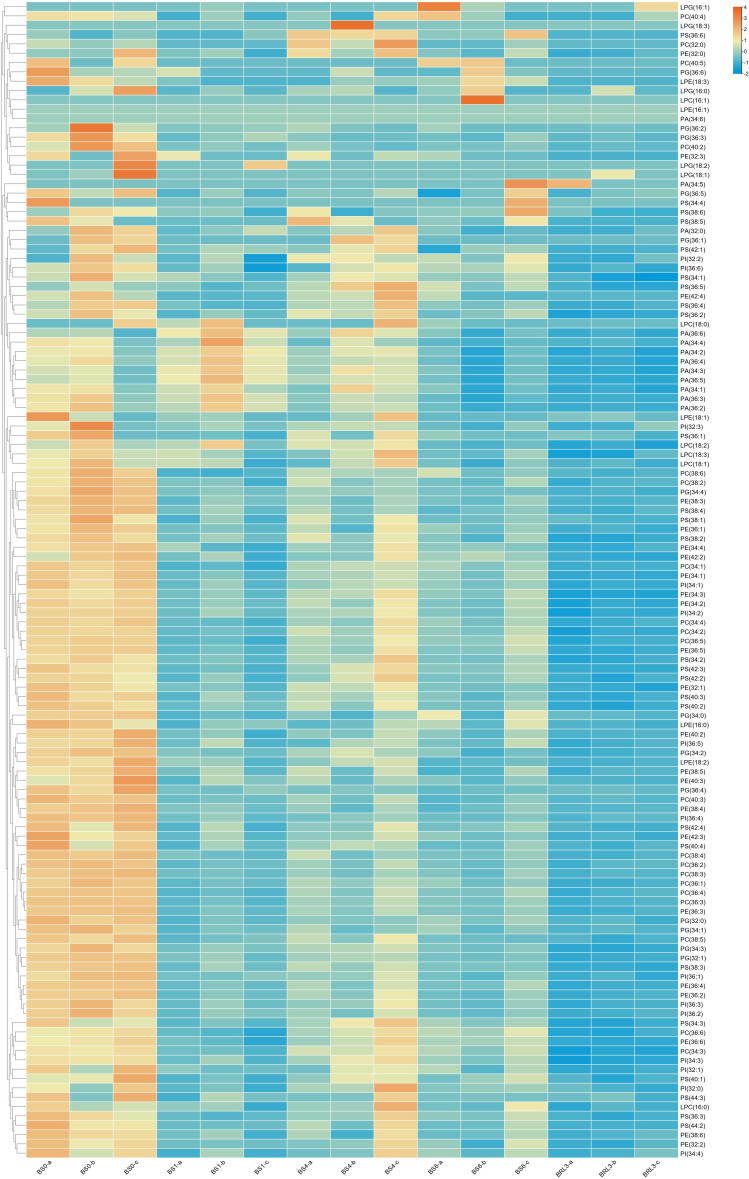
Heatmap of the changes in glycerophospholipid and lysophospholipid contents in BJG after shading and light recovery treatments. Each colored cell on the map corresponds to the content of a different lipid species. Orange color indicates a high content, and blue color indicates a low content.

Glycerophospholipids are also plant lipid components. A total of 108 glycerophospholipids were detected, which were mainly including PG, PC, PE, PI, PS and PA. As shown in **Figure 5**, comparing the overall glycerophospholipid type and contents comparison, after shading and light restoration, their changes in content were significantly different. Most phospholipids were higher in BS0, decreasing significantly after one day of shading, and the difference was the most significant after three days of light restoration. Lysophospholipids, which can be used as signaling molecules, are produced by the partial hydrolysis of phospholipids. The contents of LPC, LPG and LPE in BJG decreased dramatically to approximatlly after shading for one day.

### Analysis of significantly different lipids in BJG after shading and recovery light treatments

**Figure 6:**
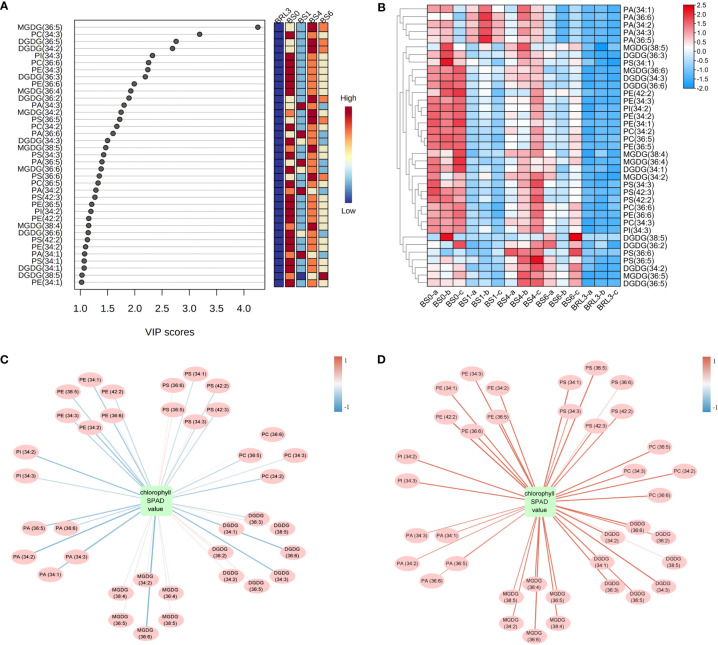
Statistical analysis of the differential lipid molecules. **(A)** Scores (VIP ≥1.0) of lipid species by PLS-DA in BJG. **(B)** Heatmap of the differential lipid changes in BJG after shading and light recovery treatments. Each colored cell on the map corresponds to the content of different lipid species. Red color indicates high, and blue color indicates low. **(C)** Correlation network between differential lipid molecules and chlorophyll SPAD value in BJG after shading. **(D)** Correlation network between differential lipid molecules and chlorophyll SPAD values in BS0 and BRL3. The red line indicates a positive correlation, the blue line indicates a negative correlation, and the darker the color is, the stronger the correlation.

After descriptive statistics of the lipids, we used VIP in the PLS-DA model to analyze the difference in BJG after shading and light recovery treatments. This value represents the contribution of each variable to the model. When VIP ≥1.0 and *P* < 0.05, lipids were considered to be significantly different. Overall, a total of 37 kinds of lipids were identified, including 6 MGDG, 8 DGDG, 2 PI, 6 PS, 4 PC, 6 PE and 5 PA (**Figure 6A**). Notably, MGDG (36:5), PC (34:3), DGDG (36:5) and DGDG (34:2) gave relatively high VIP scores. Hence, these four substances play a crucial role in BJG after shading and light recovery treatments. After constructing the heatmap, all differential lipids in BRL3 showed a significant negative correlation with the other treatments. In BS0, approximately 24 differential lipids showed a positive correlation with the other treatments, among a significant positive correlation was observed with BS1 and BRL3. We also found that in BS6 and BRL3, PA (34:1), PA (36:6), PA (34:2), PA (34:3) and PA (36:5) showed a significant negative correlation compared with BS0, BS1 and BS4 (**Figure 6B**).

Chloroplasts are not only the site of plant photosynthesis, but also the major site for fatty acid synthesis. Therefore, we mapped the correlation networks between the chlorophyll SPAD values and 37 different lipids under shading and light conditions, respectively. It can be seen from **Figure 6C** and **Figure S1A** show that under shading conditions, most differential lipids were negatively correlated with the chlorophyll SPAD value, among which PA (34:2), MGDG (36:6), DGDG (34:3) and DGDG (36:6) were significantly negatively correlated with the chlorophyll SPAD value (more blue lines). On the contrary, 24 kinds of lipids showed a significant positive correlation with chlorophyll under light conditions (more red lines). They were 5 MGDG, 5 DGDG, 2 PS, 4 PC, 2 PI and 6 PE, including MGDG (36:6), DGDG (34:3) and DGDG (36:6) (**Figure 6D** and **Figure S1B**).

### Expression patterns of key genes in lipid metabolism pathways

**Figure 7:**
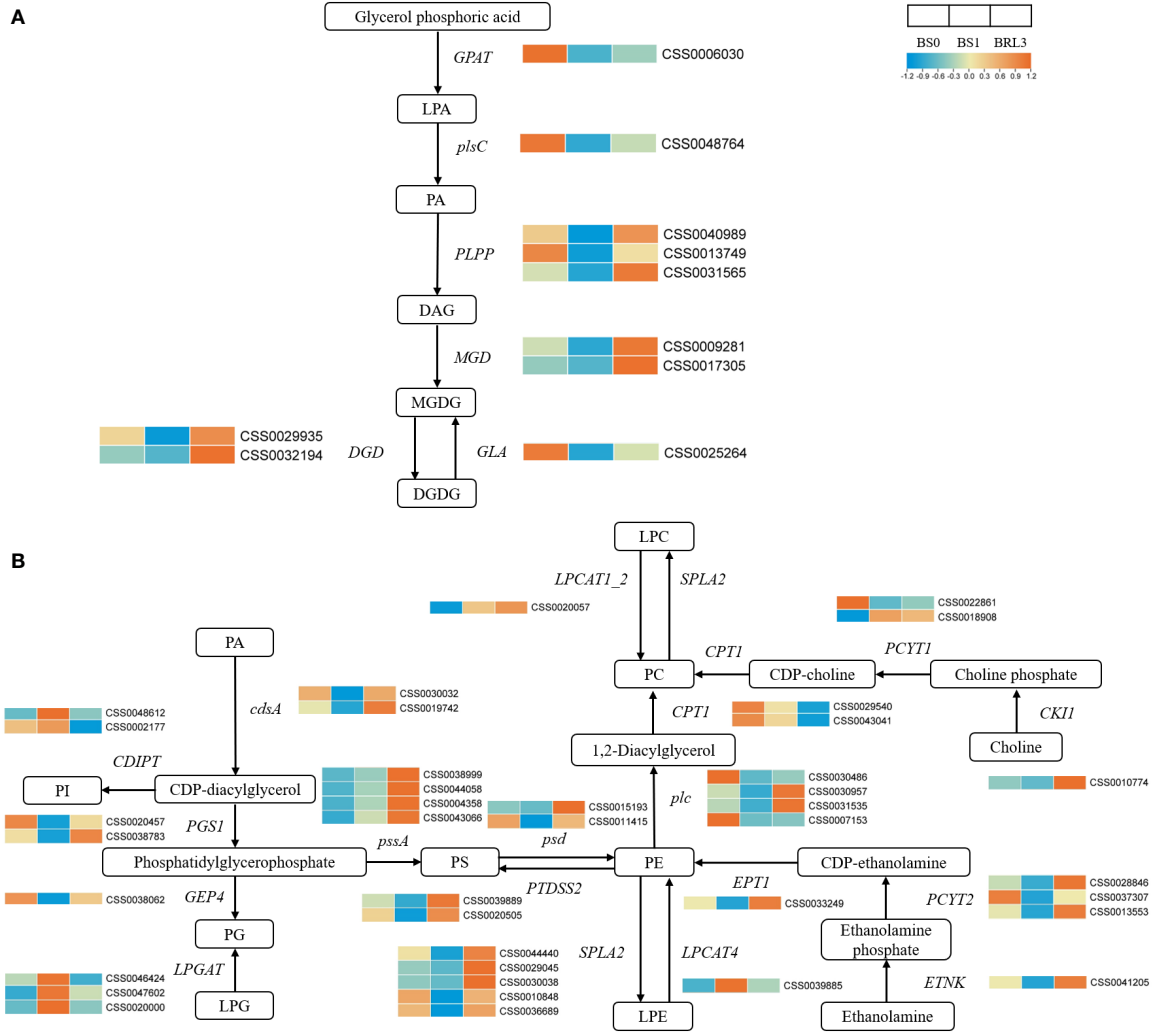
Effects of shading and light restoration on the expression of key genes in the pathway of Glycerolipid metabolism and Glycerophospholipid metabolism in BJG. **(A)** The expression of key genes in the pathway of glycerolipid metabolism. *GPAT*, glycerol-3-phosphate O-acyltransferase; *plsC*, 1-acyl-sn-glycerol-3-phosphate acyltransferase; *PLPP*, phosphatidate phosphatase; *MGD*, 1, 2-diacylglycerol 3-beta-galactosyltransferase; *DGD*, DGDG synthase; *GLA*, alpha-galactosidase. **(B)** The expression of key genes in the pathway of glycerophospholipid metabolism. *cdsA*, phosphatidate cytidylyltransferase; *PGS1*, CDP-diacylglycerol-glycerol-3-phosphate 3-phosphatidyltransferase; *GEP4*, phosphatidylglycerophosphatase; *LPGAT*, lysophosphatidylcholine acyltransferase; *pssA*, CDP-diacylglycerol-serine O-phosphatidyltransferase; *psd*, phosphatidylserine decarboxylase; *PTDSS2*, phosphatidylserine synthase 2; *SPLA2*, secretory phospholipase A2; *LPCAT4*, lysophospholipid acyltransferase; *plc*, phospholipase C; *LPCAT1_2*, lysophosphatidylcholine acyltransferase/lyso-PAF acetyltransferase; *CPT1*, diacylglycerol cholinephosphotransferase; *PCYT1*, choline-phosphate cytidylyltransferase; *CKI1*, choline kinase; *ETNK*, thanolamine kinase; *PCYT2*, ethanolamine-phosphate cytidylyltransferase; *EPT1*, ethanolaminephosphotransferase; *CDIPT*, CDP-diacylglycerol-inositol 3-phosphatidyltransferase. Orange color indicates high, and blue color indicates low.

Based on the changes in polar lipids observed during BJG shading, we focused on the expression patterns of key genes related to the lipid biosynthesis pathway. Among them, we focused on the key genes in glycerolipid metabolism and glycerophospholipid metabolism. As shown in **Figure 7A**, the genes involved in the synthesis of PA and MGDG (*GPAT*, *plsC*, *MGD*, *GLA*) were downregulated after shading and upregulated after light recovery. Genes involved in *DGD* also showed the same trend.

The metabolic pathway of glycerophospholipid is mainly involved in the synthesis of polar lipids such as PC, PE and PG. The genes involved in PG synthesis, *cdsA*, *PGS1* and *GEP4*, were downregulated in BS1 and upregulated after light recovery. *LPGAT* showed the opposite trend. The synthesis of PE is mainly involves by *ENTK*, *PCYT2*, *EPT1*, *psd* and *LPCAT4*. Except for *LPCAT4*, these genes were up-regulated under light and downregulated after shading. The same was true for the synthesis of PS, which PI was just the opposite. PC is mainly synthesized from choline and PE. Among these genes, *CPT1* showed the same trend as the other phospholipid synthetases and was downregulated after shading. However, *CKI1*, *PCYT1* and *plc* had no obvious trend. In summary, we found that the contents of polar lipids and gene expression were consistent in BS0 and BS1, and the expression of synthetic genes increased, and the lipid content also increased. However, in BRL3, the content of polar lipids was inconsistent gene expression (**Figure 7B**).

### WGCNA analysis

**Figure 8:**
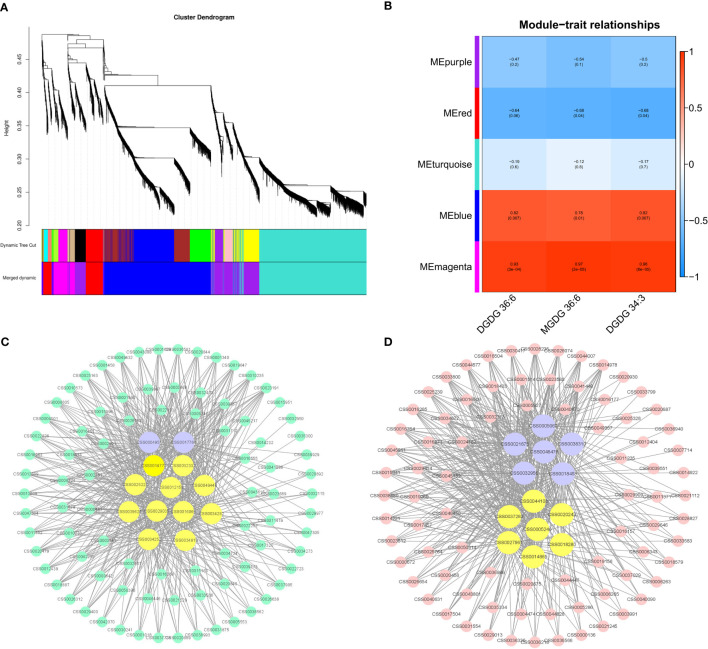
Coexpression analysis networks of lipids related to albino leaf color in BJG. **(A)** Cluster dendrogram and gene modules after WGCNA analysis. **(B)** Correlation between five gene modules and the contents of three important lipid contents. **(C)** Coexpression network of candidate genes in the Memagenta module. **(D)** Coexpression network of candidate genes in the Meblue module.

To further study the change in the pattern of membrane lipid content after BJG shading treatment, we selected DGDG (36:6), MGDG (36:6) and DGDG (34:3), which were significantly related to the chlorophyll SPAD value as phenotypic traits, and used WGCNA to search for coexpressed genes. A total of 5 modules (distinguished by different colors) were obtained, and the number of genes included in the different modules ranged from 502 to 2665. **Figures 8A, B** showed that the Memagenta module and Meblue module displayed the most significant positive correlation with the contents of these key lipids, so we conducted further analysis on these two modules.

### Functional and pathway enrichment analysis

The genes in the Memagenta and Meblue modules were analyzed for function and pathway enrichment. As shown in **Figure S2A**, pathways such as phenylpropanoid biosynthesis and glycerophospholipid metabolism are enriched in the Memagenta module. “protein processing in endoplasmic reticulum”, “phenylpropanoid biosynthesis”, “biosynthesis amino acid”, “carbon metabolism” and “flavonoid biosynthesis” were enriched in the Meblue module (**Figure S2B**).

### Lipid-related coexpressed gene networks

The top 100 unigenes (kME > 0.7) in the Memagenta module and Meblue module were used to construct the coexpression network, and transcription factors and lipid-related genes were selected as key hub genes in combination with KEGG enrichment. As shown in the **Figure 8C**, we identified 13 key hub genes in the Memagenta module, including one *Dof* TF (CSS0004951), one *ERF* TF (CSS0017764), one *ACE* gene (CSS0029035), one *GPAT* (CSS0016066), one *VPS18* (CSS0039624), 1 *OSBP* (CSS0049488) and 7 *GLIP* genes (CSS0012159, CSS0034282, CSS0014773, CSS0032332, CSS0034252, CSS0025222, CSS0034819). Similarly, in the Meblue module, we also identified 13 key hub genes. These genes included six TFs and seven lipid- related genes: one *bHLH* TF (CSS0038310), three MYB-related TFs (CSS0005060, CSS0018453, CSS0032956), one *MYB 12* TF (CSS0021675) and one *HY5* TF (CSS0048476), one *fabI* gene (CSS0044108), one *GCP6* gene (CSS0018280), one *ACE* gene (CSS0014865), one *ACS* gene (CSS0005240), one *PATL6* gene (CSS0027961), one *CXE* gene (CSS0037283) and one *ACC1* gene (CSS0020243) (**Figure 8D**). These results indicate that the lipid metabolism in BJG involves a complex molecular regulation process.

## Discussion

To date, many studies have focused on the regulatory mechanism of chlorophyll metabolism in albino tea plants, and multiomics analysis is an excellent tool for such research (Xu et al., 2020; Liu et al., 2022; Zhang et al., 2022a; Zhang et al., 2022b). However, there has been no comprehensive study on the response of lipid metabolism to light in albino tea plants. Therefore, the purpose of our research was to understand the changes in the lipid response to light in albino tea plants, and to try to determine the key pathways and genes involved in these lipid changes to make good use of the advantages of albino tea plants in tea cultivation and breeding.

### The leaf color of BJG was regulated by light intensity

The mechanism of plant leaf albinism may involve multiple regulatory pathways and metabolic processes, and may be affected by the interactions with internal and external factors (Costa et al., 2006). In higher plants, the occurrence of white or yellow leaves mainly depends on the biosynthesis and transportation of chlorophyll. Chlorophyll absorbs light energy and transfers it to other molecules in the photosynthetic electron transport chain (Liu et al., 2009). Therefore, the leaf phenotype of albino teas is closely related to chlorophyll content and is regulated by light intensity. BJG is more sensitive to high light intensity; its chloroplast development is retarded, and its chlorophyll content is lower than that of other green-leaf varieties at the same developmental stage (Wu et al., 2016). Moreover, the chlorophyll accumulation in plants is dynamic (Tian et al., 2011; Ding et al., 2016) and is a comprehensive process of synthesis and decomposition. Our studies have shown that the SPAD value of BJG leaves basically did not change in the natural environment, while increased linearly with shading treatment. The leaves notably turned green after shading treatment, while they returned to yellow if light treatment is resumed. *Huangjinya* is one of the most widely studied albino tea varieties, and shading treatment significantly increases its chlorophyll content (Fan et al., 2019). Similarly, the yellow or pale leaves of *Yujinxiang* also turned green under weak light intensity after shading, and the green leaves induced by shading recovered their yellow or pale color again by re-exposure to natural sunlight (Xu et al., 2020). This revealed that light plays a key role in regulating leaf color.

### BJG responds to light changes by changing the lipid contents and proportions

The effect of membrane lipids on the photosystem is multifaceted. When subjected to environmental stress, plants protect themselves from damage by changing the composition of membrane lipids to maintain normal growth. Under normal light, the polar lipids in BJG are generally maintained at a high level. The most important unsaturated fatty acids in DGDG and MGDG, account for a large proportion, so that albino leaves can adapt to normal light and grow to be healthy. The contents of MGDG and DGDG in cold-tolerant pepper varieties were higher than those in low-temperature-sensitive varieties, which could better maintain the membrane homeostasis (Xu et al., 2021). Plants maintain a balance between the physical state of the membrane and the normal function of membrane proteins by adjusting the ratio of MGDG/DGDG (Narasimhan et al., 2013). When the ratio is high, the membrane stability is better and the thylakoid membrane integrity is higher; when the ratio is low, the thylakoid membrane structure is unstable. The ratio of MGDG/DGDG in albino leaves was low, and the thylakoid membrane structure was unstable. After shading, the leaf color gradually turned green, and the ratio suddenly rose. The thylakoid membrane tends to be complete at first, and then its structure becomes structure is unstable after the light is removed. This means that BJG can respond quickly to changes in light. After the restoration of light, the ratio was similar to that under normal light conditions, indicating that the thylakoid membrane structure remained a relatively stable state at this time. However, the ratios of membrane lipid/chlorophyll (MGDG/chlorophyll and DGDG/chlorophyll), which are indicators of thylakoid assembly protein density, decreased with increasing treatment time, indicating that the thylakoid assembly protein density increased from low to high, and the membrane structure gradually stablized. This is consistent with a study in peppers (Xu et al., 2021). This stability may represent an adaptive strategy that enables plants to tolerate stress by maintaining cell membranes in a physical state that supports the normal functions of membrane proteins. According to the correlation analysis, we discovered three key lipid components that are significantly related to the chlorophyll SPAD value, which are MGDG (36:6), DGDG (36:6) and DGDG (34:3), which are found that they are thylakoid membrane lipids with high contents. This further suggested that membrane lipids can respond to light and promote the color changes in light-sensitive albino tea leaves.

### The genes *HY5* and *GLIP* can participate in the lipid regulation of albino tea leaves

Changes in the composition, contents and proportions of membrane lipids will lead to changes in the integrity of the thylakoid membrane, and genes in the lipid metabolism pathway can respond over time. To explore the mechanism of the lipid response to leaf color changes in light-sensitive albino tea plants, we performed transcriptome sequencing of BJG leaves exposed to different light treatments. Lipid metabolism is regulated by many genes and TFs (Afitlhile et al., 2015; Afitlhile et al., 2021). An increase in membrane lipid synthesis gene expression can promote membrane lipid synthesis, thus allowing the thylakoid membrane structure to be intact. Genes involved in the synthesis of PA, MDGD and DGDG (*GPAT*, *PLSC*, *MGD*, *DGD*, *GLA*) were downregulated after shading and upregulated after light recovery, which indicated that light could promote the synthesis of lipids in albino leaves, while shading had the opposite effect. This also explains why the lipid content in BS0 was higher but significantly decreased after shading. Similar results have been confirmed in Arabidopsis. *MGD1* can be upregulated by light, and the expression of *MGD1* in etiolated seedlings of wild-type Arabidopsis thaliana increased twofold after 6 hours of light exposure. The expression of *DGD1* in wild-type seedlings increased after illumination, while the expression of *DGD2* remained unchanged (Kobayashi et al., 2014). However, the expression of synthetic genes is not consistent with their contents after light recovery, so we speculate that lipid degradation genes may be involved. The genes involved in PG synthesis, *cdsA*, *PGS1* and *GEP4*, were downregulated in BS1 and upregulated after light recovery. *LPGAT* displayed the opposite trend. Therefore, we speculate that change in PG content after shading is mainly caused by the degradation of LPG.

In this study, we found 8 TFs and 18 hub genes based on WGCNA, which may be related to lipid metabolism. The TFs were one *Dof* (CSS0004951), one *ERF* (CSS0017764), one *bHLH* TF (CSS0038310), three *MYB-related* TFs (CSS0005060, CSS0018453, CSS0032956), one *MYB 12* TF (CSS0021675) and one *HY5* TF (CSS0048476). *Dof* TFs are a family of plant-specific transcription factors (Yanagisawa, 2002). It has been reported that proteins containing the Dof domain are involved in many different plant-specific physiological processes, including light-dependent gene regulation in maize (Yanagisawa and Sheen, 1998). *HY5*, a transcription factor of the *bZIP* class, is located downstream of photoreceptors and transmits light signals to downstream acting-elements. Under light and dark changes, the dynamic processes of *HY5*, *COP1* and *ABI4* in the nucleus and cytoplasm regulate the key genes of many tetrapyrrole synthetases, such as *PORA*, *HEMA2* and *FC2* (Burko et al., 2020; Hand and Shabek, 2022). Therefore, this will affect the chlorophyll biosynthesis process. In addition, *HY5* also targets many genes involved in lipid biosynthesis, such as *DGD1*, *FAD3*, and *FAD6*. Genome-wide analysis has identified that *DGD1* and *CHLH* are direct targets of *HY5* (Lee et al., 2007). Similar studies have also found that during the process of photomorphogenesis, light signals through *HY5* and cytokinin signals through *AHK2* and *AHK3* were involved in the up regulation of *MGD1* and *DGD1* (Kobayashi et al., 2014). In our research, the expression of *HY5* was significantly downregulated after shading and upregulated after light recovery (**Figure S3**), showing the same expression trend as lipid-related genes such as *DGD*, which further confirmed that *HY5* was closely related to lipid biosynthesis.

*GPAT* was identified among the 18 hub genes. This is an important group of enzymes that catalyzes the acylation of sn-glycerol-3-phosphate at sn-1 or sn-2 to produce lysophosphatidic acid. This reaction is the first step in the assembly of stored lipids and is also important in polar and extracellular lipid biosynthesis (Jayawardhane et al., 2018). For example, overexpression of *SsGPAT* in the halophyte *Suaeda salsa* in Arabidopsis leads to enhanced salt tolerance, which may be to alleviate the photoinhibition of PSII and PSI under salt stress by increasing the content of unsaturated fatty acids (Sui et al., 2017). Similarly, in this study, the expression of *GPAT* under natural light was higher than that under shading, which could also confirm its contribution to alleviating photoinhibition.

In particular, we found 7 *GLIP* genes (CSS0012159, CSS0034282, CSS0014773, CSS0032332, CSS0034252, CSS0025222, CSS0034819), which are the members of a subfamily of lipolytic enzymes with broad substrate specificity that have been widely identified in plants (Gao et al., 2017). Physiologically, the GDSL esterase/lipase found is mainly involved in the regulation of plant development, morphogenesis, secondary metabolite synthesis and defense response (Oh et al., 2005; Kwon et al., 2009; Agee et al., 2010). The comprehensive lipid profiling analysis of rice by some researchers showed that the *OsGLIP* gene negatively controls rice defense by regulating lipid metabolism. Changes in *OsGLIP* expression is related to the significant changes in lipid species (including MGDG and DGDG), which are most likely to be inhibitors of the immune response in rice. Exogenous addition of MGDG and DGDG can reduce disease resistance (Gao et al., 2017). In our study, the expression of *GLIP* genes in BS0 was significantly higher than that in BS1 and BRL3, and the contents of MGDG and DGDG were also maintained at a high level (**Figure S3**). Therefore, this result can also explain why the stress resistance in albino leaves is poor, and provide a new idea for tea breeding.

## Conclusion

Our study compared the effects of light intensity on the lipid metabolites and transcripts in albino tea plants, and found that the leaf color of BJG was regulated by light intensity and responded to light changes by changing the lipid contents and proportions. Three lipids that were significantly related to the chlorophyll SPAD value were found among the differential lipids: MGDG (36:6), DGDG (36:6) and DGDG (34:3). The results of WGCNA analysis indicated that the *HY5* and *GLIP* genes may be hub genes involved in lipid regulation in albino leaves. These results can further explain the changes in tea leaf color in response to light. Therefore, the future research can focus on explaining the interaction between these hub genes and TFs, with an aim to provide a reference for genetic breeding and research on albinism mechanisms.

## Data availability statement

The data presented in the study are deposited in the OMIX, China National Center for Bioinformation/Beijing Institute of Genomics, Chinese Academy of Sciences, accession number OMIX002015.

## Author contributions

WS, MC, ZC, and QW conceived and designed the experiments; ZZ performed the experiments; ZZ analyzed the data; ZZ wrote the manuscript; and MC, QW, and WZ revised the manuscript critically. WS and ZC oversaw the project. All the authors read and approved the final manuscript.

## Funding

This work was financially supported by the Fujian Agriculture and Forestry University Construction Project for Technological Innovation and Service System of Tea Industry Chain (K1520005A01/K1520005A04), the National Natural Science Foundation of China (31770732), the cooperative project of production and study in universities of Fujian Province (2019N5007), Special Fund for Science and TechnologyInnovation of Fujian Zhang Tianfu Tea Development Foundation (FJZTF01).

## Acknowledgments

Thanks are extended to Shixian Cao and Shuntian Yu for support in sample gathering, and the Kansas Lipidomics Research Center for lipid analysis.

## Conflict of interest

The authors declare that the research was conducted in the absence of any commercial or financial relationships that could be construed as a potential conflict of interest.

## Publisher’s note

All claims expressed in this article are solely those of the authors and do not necessarily represent those of their affiliated organizations, or those of the publisher, the editors and the reviewers. Any product that may be evaluated in this article, or claim that may be made by its manufacturer, is not guaranteed or endorsed by the publisher.
