# Supplementary material for: Combined analysis of lipidomics and transcriptomics revealed the key pathways and genes of lipids in light-sensitive albino tea plant (Camellia sinensis cv. Baijiguan)
Source: Front Plant Sci. 2022 Oct 18;13:1035119. doi: 10.3389/fpls.2022.1035119 (PMC9623167; doi:10.3389/fpls.2022.1035119)
Supplement: Supplementary file 1 [file DataSheet_1.docx]

Supplementary Material


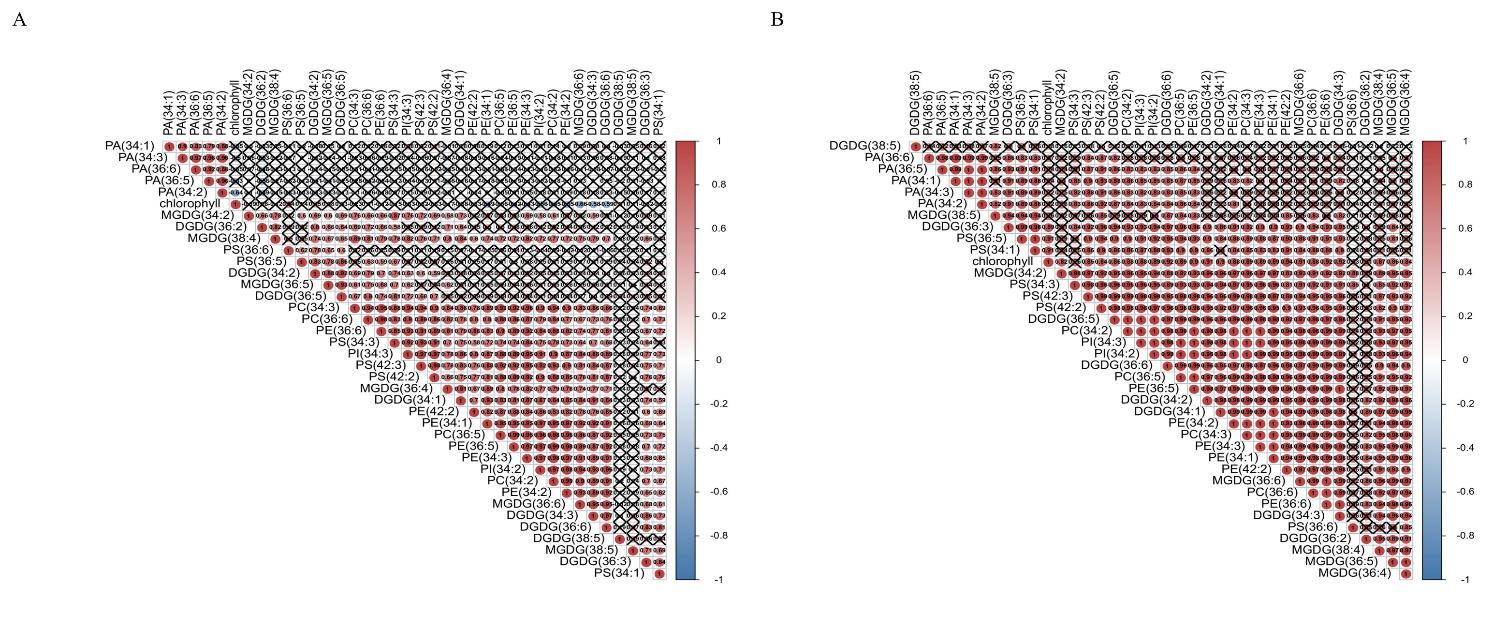


**Fig. S1** Correlation analysis between differential lipid molecules and chlorophyll SPAD value. (A) Correlation analysis between differential lipid molecules and chlorophyll SPAD value of BJG after shading. (B) Correlation analysis between differential lipid molecules and chlorophyll SPAD value in BS0 and BRL3. Red color indicates high, and blue color indicates low.


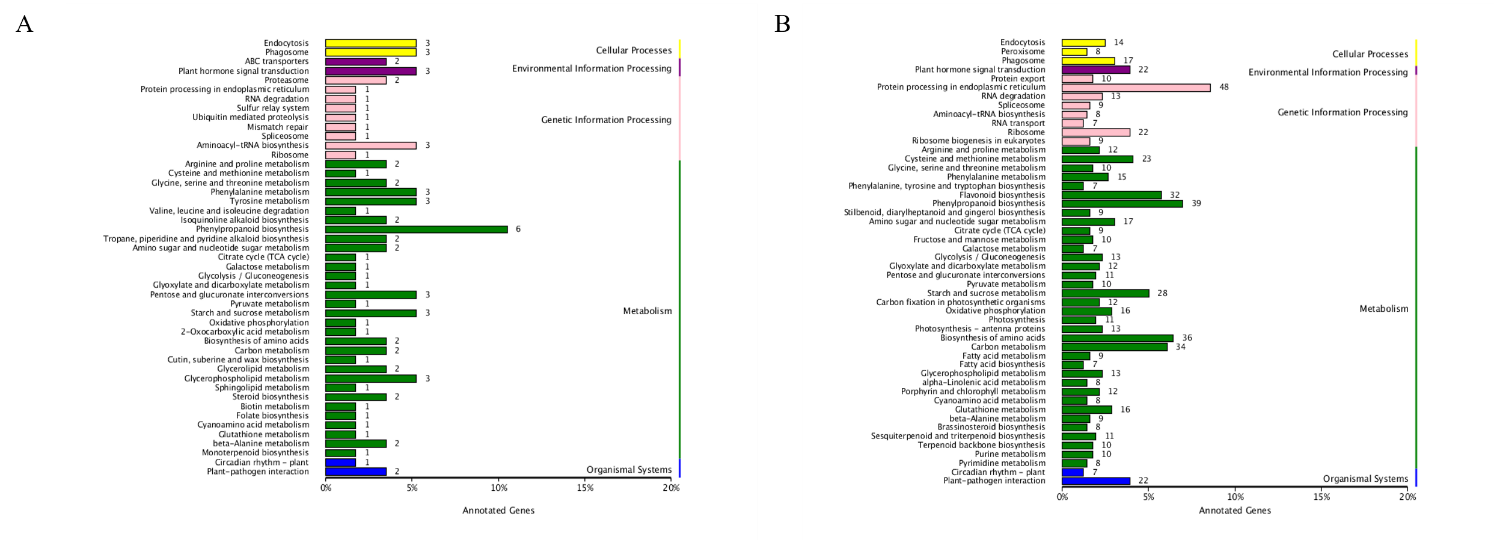


**Fig. S2** KEGG classification diagram of the Memagenta and Meblue modules. (A) KEGG classification diagram of the Memagenta module. (B) KEGG classification diagram of the Meblue module.


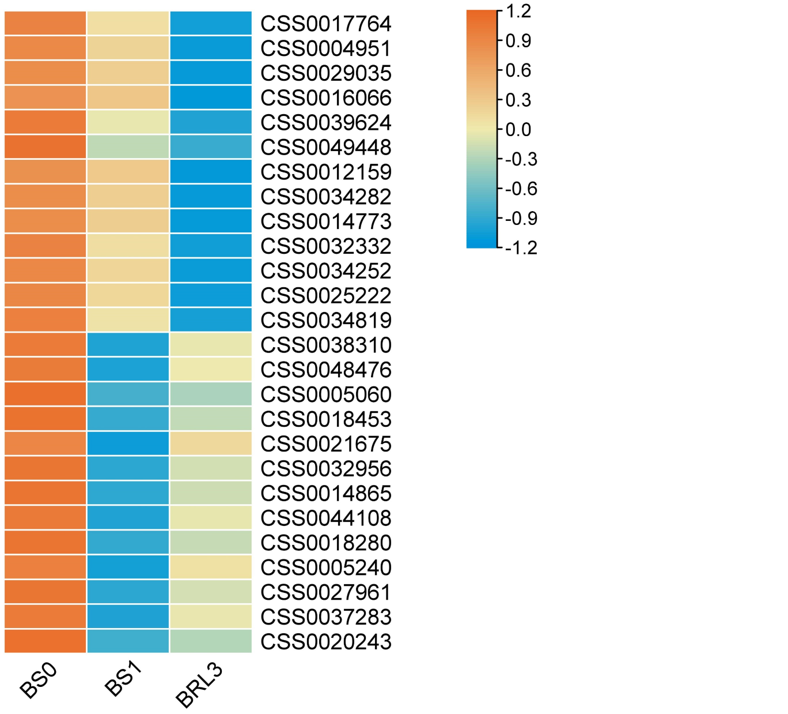


**Fig. S3** Heatmap of FPKM values of hub genes. Orange color indicates high, and blue color indicates low.

**Table S1** The total contents of Glycolipids in BJG after shading and recovering light treatments (nmol/mg dw)

| Classification | Compounds name | Compounds | Mass | BS0 | BS1 | BS4 | BS6 | BRL1 |
| --- | --- | --- | --- | --- | --- | --- | --- | --- |
|  | DGDG (34:4) | C_49_H_84_O_15_ | 930.6 | 0.09±0.01 a | 0.04±0.02 b | 0.06±0.01 b | 0.04±0.01 b | 0.01±0 c |
|  | DGDG (34:3) | C_49_H_86_O_15_ | 932.6 | 5.23±0.37 a | 2.41±0.29 c | 3.89±0.21 b | 2.43±0.31 c | 1.27±0.10 d |
|  | DGDG (34:2) | C_49_H_88_O_15_ | 934.6 | 0.21±0.02 a | 0.12±0 b | 0.28±0.05 a | 0.24±0.06 a | 0.08±0.01 b |
|  | DGDG (34:1) | C_49_H_90_O_15_ | 936.6 | 0.40±0.05 a | 0.18±0.03 cd | 0.29±0.03 b | 0.23±0.07 bc | 0.11±0.02 d |
|  | DGDG (36:6) | C_51_H_84_O_15_ | 954.6 | 5.71±0.55 a | 2.53±0.49 c | 3.88±0.42 b | 2.40±0.34 c | 1.25±0.09 d |
|  | DGDG (36:5) | C_51_H_86_O_15_ | 956.6 | 0.32±0.11 a | 0.19±0.03 b | 0.39±0.12 a | 0.33±0.05 a | 0.11±0.01 b |
| DGDG | DGDG (36:4) | C_51_H_88_O_15_ | 958.6 | 0.73±0.05 a | 0.30±0.06 b | 0.41±0.01 c | 0.32±0.05 c | 0.14±0.01 d |
|  | DGDG (36:3) | C_51_H_90_O_15_ | 960.6 | 1.25±0.24 a | 0.66±0.07 b | 1.10±0.16 a | 0.78±0.11 b | 0.31±0.02 c |
|  | DGDG (36:2) | C_51_H_92_O_15_ | 962.6 | 0.07±0.03 a | 0.02±0 b | 0.07±0.01 a | 0.07±0.03 a | 0.02±0.01 b |
|  | DGDG (36:1) | C_51_H_94_O_15_ | 964.7 | 0.05±0.03 a | 0.03±0 ab | 0.04±0.01 a | 0.02±0.01 ab | 0.01±0 b |
|  | DGDG (38:6) | C_53_H_88_O_15_ | 982.6 | 0.10±0.02 a | 0.02±0.01 b | 0.07±0.02 a | 0.01±0 b | 0.01±0 b |
|  | DGDG (38:5) | C_53_H_90_O_15_ | 984.6 | 0.01±0 | N.D. | N.D. | 0.01±0 | N.D. |
|  | DGDG (38:4) | C_53_H_92_O_15_ | 986.6 | 0.04±0 a | 0.01±0 b | 0.01±0 b | 0.01±0 bc | N.D. |
|  | DGDG (38:3) | C_53_H_94_O_15_ | 988.7 | 0.01±0 | N.D. | N.D. | N.D. | N.D. |
|  | MGDG (34:6) | C_43_H_70_O_10_ | 764.5 | 0.02±0 a | N.D. b | 0.01±0 b | N.D. b | N.D. b |
|  | MGDG (34:5) | C_43_H_72_O_10_ | 766.5 | 0.01±0 a | 0.01±0 a | 0.01±0 a | N.D. b | N.D. b |
|  | MGDG (34:4) | C_43_H_74_O_10_ | 768.5 | 0.19±0.05 a | 0.09±0.02 b | 0.12±0.01 b | 0.06±0.01 bc | 0.02±0 c |
|  | MGDG (34:3) | C_43_H_76_O_10_ | 770.5 | 1.22±0.21 a | 0.69±0.15 b | 0.73±0.21 b | 0.44±0.04 bc | 0.32±0.05 c |
|  | MGDG (34:2) | C_43_H_78_O_10_ | 772.6 | 0.07±0.02 a | 0.04±0.01 b | 0.08±0.01 a | 0.05±0.02 ab | 0.01±0 c |
| MGDG | MGDG (34:1) | C_43_H_80_O_10_ | 774.6 | 0.03±0 a | N.D. b | 0.01±0 b | 0.01±0 b | 0.01±0 b |
|  | MGDG (36:6) | C_45_H_74_O_10_ | 792.5 | 19.56±2.40 a | 9.83±1.03 c | 13.69±1.02 b | 8.06±1.45 c | 4.47±0.18 d |
|  | MGDG (36:5) | C_45_H_76_O_10_ | 794.5 | 1.49±0.23 a | 0.90±0.16 b | 1.88±0.50 a | 1.58±0.22 a | 0.50±0.04 b |
|  | MGDG (36:4) | C_45_H_78_O_10_ | 796.6 | 0.99±0.21 a | 0.42±0.03 c | 0.69±0.11 b | 0.69±0.10 b | 0.26±0.02 c |
|  | MGDG (36:3) | C_45_H_80_O_10_ | 798.6 | 0.29±0.03 a | 0.15±0.03 bc | 0.20±0.03 b | 0.13±0.02 cd | 0.08±0 d |
|  | MGDG (36:2) | C_45_H_82_O_10_ | 800.6 | 0.02±0 a | 0.01±0 abc | 0.01±0 bc | 0.02±0 ab | N.D. c |
|  | MGDG (38:6) | C_47_H_78_O_10_ | 820.6 | 0.13±0.03 a | 0.07±0.02 bc | 0.07±0.02 b | 0.03±0.01 cd | 0.02±0.01 d |
|  | MGDG (38:5) | C_47_H_80_O_10_ | 822.6 | 0.04±0.02 a | 0.03±0 ab | 0.04±0.01 a | 0.04±0.01 a | 0.01±0 b |
|  | MGDG (38:4) | C_47_H_82_O_10_ | 824.6 | 0.02±0 a | 0.01±0 bc | 0.02±0 a | 0.01±0 b | N.D. c |

*Note:* Data and error bars are the mean ± SD (n = 3). N.D.: not detect. Different lowercase letters indicate a significant difference between the means at *P*<0.05.
